# Progress and Policy

**Published:** 1918-10-19

**Authors:** 


### THE MATRONS' AND SISTERS' DEPARTMENT.

### PROGRESS AND POLICY.

Lord Grey, in his recent illuminating speech on the League of Nations, made a very interesting comparison of the League to a steam engine, wherein motive force and guiding intelligence were of no avail until the machinery had been perfected. The comparison aptly fits the College of Nursing. Its machinery consists of its constitution, its business backbone, its methods of government in London and the Provinces. Its motive power lies in the union of nurses who give it their support. Both are indispensable, and we have devoted some space to a detailed consideration of the manner in which the machine has been constructed in order that it may gather to itself the forces necessary for motion. But, after all, every one is agreed that the really vital matter is the direction in which this engine moves. Is the College to lend the nursing profession one more arid example of endless committee meetings, elections, discussions, and heart-burnings? Or has it definite objects to attain, a definite policy of reconstruction in view attainable in no other way?

A great deal of nonsense is being talked about the desirability of nurses managing their own affairs, and an attempt is being made to screw down the application of the word "nurse" to an imaginary body of women unconnected with any governing body. It cannot be too clearly recognised that nurses as a body in this sense simply do not exist. Nurses form a stream rather than a body. They rarely enter the profession and remain independent entities, as a doctor does, for instance, once he is fully qualified. They enter as subalterns, but they do not remain subalterns. They pass on to a multiplicity of posts, in all of which they both exercise authority and submit to it. Those who have ideas rise naturally to positions in which their ideas become fruitful for others. United they form a body of immense power. Sorted out into supposititious categories of "matrons" and "nurses," mutually distrustful and opposed each to the interests of the other, they become merely an agglomeration of women working for their own hands. Nothing could be further from the truth.

The vast majority of trained nurses reject and repudiate with scorn the notion of being oppressed and down-trodden by the matrons, committees, governors, and what not under whom they work. On the contrary, we find the most cordial relations subsisting among the various degrees of nurses in the training-schools and nursing institutions all over the country. All but the youngest probationers are in positions of responsibility varying in degree, and are conscious of being part of a slowly-moving stream which accomplishes its work in unison. Part of this stream is for ever passing out into the larger world, but not for that does the nurse lose her sense of the unity with which her training-school endowed her. She belongs to it for life. The impulse it imparted forms the guiding force of her professional career. She carries its message to the slums, to the lonely village, to the furthest corners of the Empire. The training-schools do, in fact, represent the coherent professional opinion of that great stream of nurses which waters the earth.

We have had nursing machinery before now, some of it quite admirable in ingenuity. We have never before had the motive force bestowed by the adhesion of 12,000 nurses. Most of all, we have lacked the guiding intelligence which only the united minds of the training-schools could supply. From now on we watch the engine, with steam up and wisely guided, begin to move off towards a definite goal.

